# Whole blood gene expression within days after total-body irradiation predicts long term survival in Gottingen minipigs

**DOI:** 10.1038/s41598-021-95120-5

**Published:** 2021-08-05

**Authors:** Sunita Chopra, Maria Moroni, Jaleal Sanjak, Laurel MacMillan, Bernadette Hritzo, Shannon Martello, Michelle Bylicky, Jared May, C. Norman Coleman, Molykutty J. Aryankalayil

**Affiliations:** 1 National Cancer Institute (NCI), National Institutes of Health, Bethesda, MD 20892 USA; 2 Armed Forces Radiobiological Research Institute, Bethesda, MD 20889 USA; 3 Gryphon Scientific, Takoma Park, MD 20912 USA; 4 Radiation Oncology Branch, Center for Cancer Research, National Cancer Institute (NCI), Bethesda, MD 20892 USA

**Keywords:** Predictive markers, Transcriptomics, Bioinformatics, Predictive markers

## Abstract

Gottingen minipigs mirror the physiological radiation response observed in humans and hence make an ideal candidate model for studying radiation biodosimetry for both limited-sized and mass casualty incidents. We examined the whole blood gene expression profiles starting one day after total-body irradiation with increasing doses of gamma-rays. The minipigs were monitored for up to 45 days or time to euthanasia necessitated by radiation effects. We successfully identified dose- and time-agnostic (over a 1–7 day period after radiation), survival-predictive gene expression signatures derived using machine-learning algorithms with high sensitivity and specificity. These survival-predictive signatures fare better than an optimally performing dose-differentiating signature or blood cellular profiles. These findings suggest that prediction of survival is a much more useful parameter for making triage, resource-utilization and treatment decisions in a resource-constrained environment compared to predictions of total dose received. It should hopefully be possible to build such classifiers for humans in the future.

## Introduction

With radiological sources being used for diverse applications from generation of electricity, medical diagnosis and therapy, there is potential for accidental total-body or partial-body exposure. Additionally, there is the threat of radiological material misuse in the form of nuclear weapons resulting in deliberate exposure to radiation. The extant circumstances have renewed interest in the field of radiation biodosimetry for guiding clinical care should accidental or intentional exposures occur^1,2^. There is no FDA approved assay yet for use in such scenarios. The ‘chromosomal dicentric assay’ is often considered the current “gold standard” for estimating radiation dose absorbed following total-body or extensive partial-body irradiation exposure. Even with recent significant technical advances and automation, the assay requires significant expertise and the availability of viable lymphocytes for culturing^3–5^. Additionally, there is a time-lag before initial results are obtained, potentially missing an early window (12 or 24 h) for the use of radiation injury mitigators^6^. In a mass casualty scenario involving radiation exposure of a large population, simpler, easier, and quicker assays are needed for faster triaging of victims. ‘Lab-on-a-chip’ devices are being developed for assessing radiation exposure^7,8^ and triaging populations for life-threatening viral infections such as Ebola^9–12^. Nucleic acids are favored as assayable molecules in field deployable devices due to their stability and ease of identification in whole blood samples^13,14^. We undertook the present study to identify dose- and time-dependent gene expression profile changes in whole blood samples of Gottingen minipigs after total body irradiation. A predictive signature might enable development of a field deployable ‘lab-on-a-chip’ assay for rapid triaging of victims by radiation biodosimetry level. The ability to make early decisions for proper treatment and for more efficient use of scarce resources can potentially save many lives in a mass casualty scenario^15^.

Rodent models are the preferred choice for radiation animal studies due to low cost, easy handling, ability to assess multiple conditions and shorter reproductive cycles. However they do not make good candidates for biodosimetry studies because of smaller blood volume available for analysis^16–20^. A remarkable study published recently has identified a miRNA-based assay for estimating radiation dose absorbed from mice studies, which was capable of predicting radiation dose absorbed in human patient subjects^21^. However, it is almost impossible to design time course studies in mice and they do not display symptoms such as emesis seen in humans following radiation exposure. Non-human primates (NHP) make highly relevant animal models being evolutionarily closest to humans; however, ethical concerns, cost, longer reproductive cycles, and availability limit their utility. The Gottingen minipig is an alternative large animal model for studying acute radiation syndrome (ARS) and for use in testing novel radiation countermeasures ^22–24^.

The anatomy and physiology of minipigs are very similar to humans and they display similar hematopoietic ARS symptoms to those observed in humans and other large animal models (canines and NHP)^22,25^. The ileocutaneous anastomosis model developed in Gottingen minipigs has shown that radiation induced gastrointestinal ARS in minipigs mimics changes in the human GI syndrome^26^. Several bio-indicators predictive of human conditions have been found to also predict survival in total-body irradiated minipigs, such as decreased lymphocyte/granulocyte ratios, increased levels of citrulline, alkaline phosphatase, and C-reactive protein^27^. In response to radiation, circulating levels of interleukin-18 are induced in humans and were also found to be elevated in minipigs and NHP^28^. In the ɣ-H2AX-based biodosimetry assay, similarities have been observed between radio-sensitivity of lymphocytes and fibroblasts of human and porcine origins^24^. These findings illustrate the similarities in radiation response of minipigs and humans and potential usefulness of minipigs as alternate large animal models for radiation biodosimetry research.

Our laboratory recently identified organ-inherent changes in heart, lung, and liver tissue gene expression correlating with survival history of these animals^29^. In the present study, we investigated radiation-responsive mRNA expression profiles in whole blood RNA samples from total-body irradiated Gottingen minipigs for dose and survival prediction. Our findings demonstrate that blood-based gene expression signatures could predict survival in minipigs.

## Results

### Animal survival depended on the total dose received

**Figure 1:**
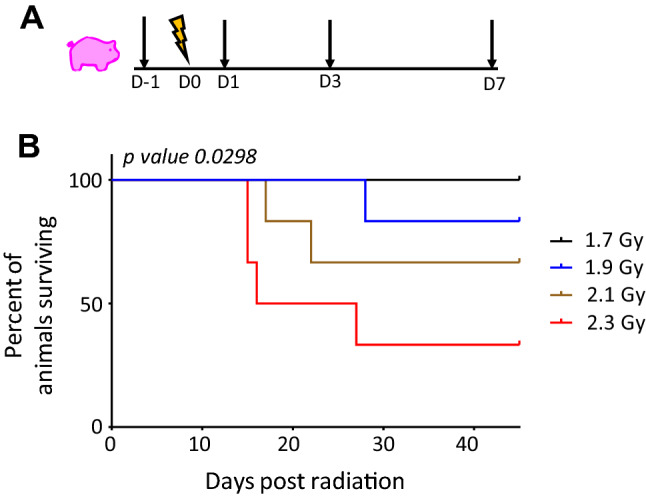
(**A**) Experimental design for blood collection for gene expression analysis. Blood was collected one day prior to irradiation (denoted as D-1); radiation day (D0), post irradiation collection 1 day (D1), 3 days (D3) and 7 days (D7) after radiation; (**B**) Kaplan Meier (K–M) Survival Curve for the animals studied. Time is plotted along x-axis and percent survival along y-axis; plot was generated in GraphPad Prism.

Figure 1A shows the experimental design for collection of blood samples subjected to gene expression analysis. Clinical signs and hematology for the irradiated minipigs were followed for 45 days. During that period, if any animal developed acute conditions and pain, it was sacrificed. The survival at day 45 for the 1.7, 1.9, 2.1, and 2.3 Gy irradiation doses was 6/6, 5/6, 4/6, and 2/6, respectively. All the decedent animals survived between days 14 and 28 post-irradiation as shown on the Kaplan–Meier curve (Fig. 1B). Hence, the doses 1.7 Gy, 1.9 Gy, 2.1 Gy and 2.3 Gy corresponded to LD_0/45_, LD_17/45_, LD_33/45_, and LD_66/45_, respectively, in this study.

### Blood cellular profiles indistinguishable between survivors and decedents up to seven days after irradiation

**Figure 2:**
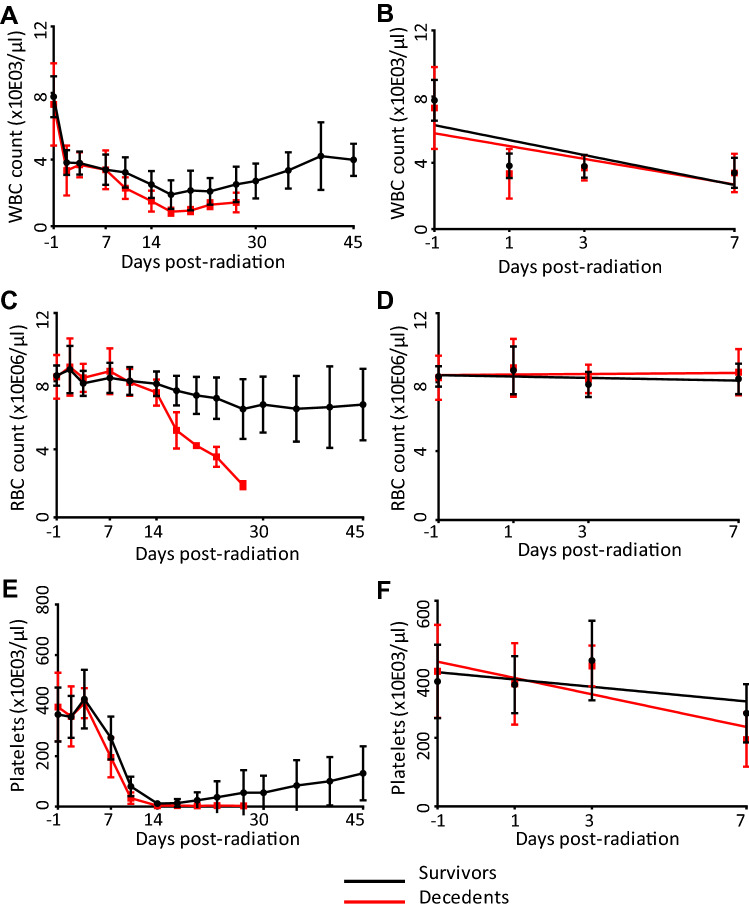
Blood parameters as a function of time in survivors and decedents. Sharp decline was observed in both decedents (red) and survivors (black) over the 45-day period for (**A**) WBC (p < 0.0001), (**C**) RBC (p < 0.0001), and (**E**) platelet counts (p < 0.0001). No significant differences were observed in the decedents and survivors over the 7-day period immediately after radiation in WBC (**B**), RBC (**D**) and platelet (**F**) counts.

Complete blood counts including white blood cells, red blood cells, and platelet counts were recorded in all the animals periodically for 45 days (supplementary Table 1). A decline is observed in all parameters over the 45-day period; in survivors we observe a recovery after an initial nadir in all parameters but not in decedents suggesting that decedents could not recover from the initial radiation injury (Fig. 2A,C,E). Interestingly, no difference is discernable between decedents and survivors during the seven days post-irradiation as is evident from overlapping curves (Fig. 2B,D,F). Neutrophil counts also followed a similar trend (Supplementary Fig. 1). Lymphocyte:neutrophil (L/N) ratio is widely believed to correlate to the dose absorbed ^30^. In the comparison between survivors and decedents, L/N ratio did not change much over the 45-day period in the survivors, but a sharp increase was observed in the decedent animals (Supplementary Fig. 2A). Over the 7-day period immediately following irradiation, no difference could be observed between decedents and survivors based on the L/N ratio (Supplementary Fig. 2B).

### Differential gene expression profiles observed in response to radiation in blood samples of TBI Gottingen minipigs

**Figure 3:**
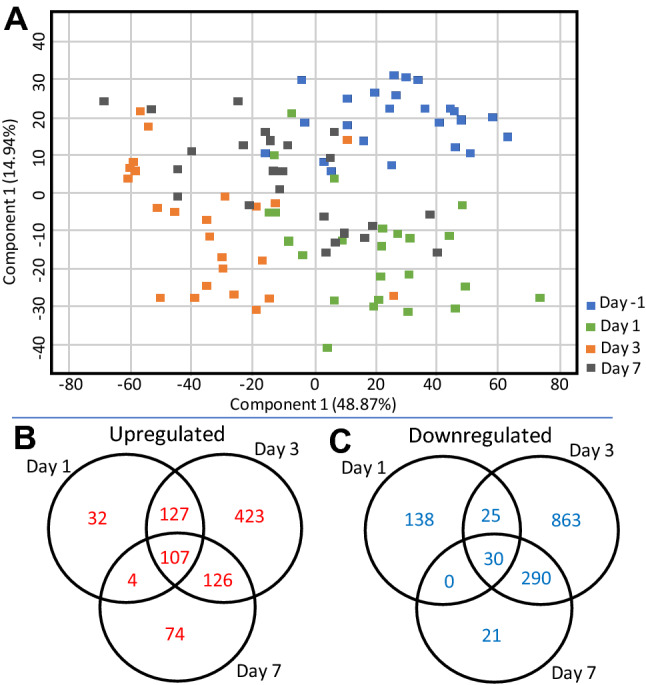
Gene expression changes most pronounced at D3 compared to baseline values. Significantly differentially expressed genes across all animals, irrespective of dose, were identified by repeated measures ANOVA and Benjamini Hochberg FDR correction (p < 0.001) followed by twofold cut-off. (**A**) PCA plot was generated in GeneSpring using the differentially regulated probe-list. (**B**) Venn diagram showing distribution of upregulated genes passing the filtering criteria at different time-points. (**C**) Venn diagram showing distribution of downregulated genes passing the filtering criteria at different time-points.

**Figure 4:**
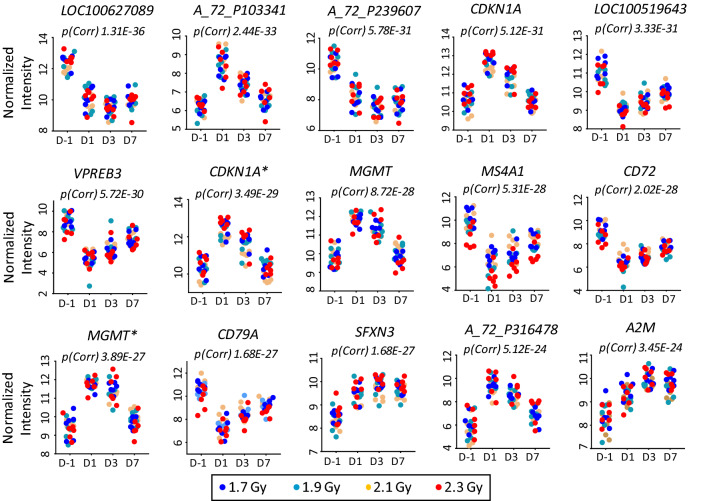
Scatter plots of the 15 most highly significantly regulated genes across all animals. The time-points of data collection are on the x-axis. D-1, D1, D3, D7 depict the day-1, day 1, day 3 and day 7, respectively. The normalized intensity values for each animal are plotted on the y-axis. *Signifies different probe for the same mRNA. Data points for 1.7 Gy, 1.9 Gy, 2.1 Gy and 2.3 Gy are depicted in dark blue, cyan, orange, and red, respectively.

Repeated measures ANOVA and Bejamini Hochberg FDR correction were applied to find differentially expressed genes along time across all the doses combined. Of the 7485 genes that were below the p-value cut-off (< 0.001), the expression of 2253 genes were regulated at least more than two-fold at any of the D1, D3 or D7 time-points compared to pre-irradiation values at D-1 (Table 1 and supplementary Table 2). Gene expression observed at D3 was most divergent from D-1 expression as observed in the PCA analysis (Fig. 3A). Subsequently, the largest gene expression differences from D-1 expression were also observed at D3 (Fig. 3B,C). The fifteen most significantly differentially expressed genes at any of the time-points irrespective of the dose are shown in Fig. 4. Total number of genes that passed stepwise filtering criteria along different doses and survivors and decedents separately are listed in Table 1. Principal component analysis (PCA) performed on these lists also show greatest separation between D-1 and the D3 time points (Supp. Figure 3, 4A, B). Consequently, compared to D-1, D3 showed the largest number of genes differentially expressed in both decedents and survivors (Supp. Figure 4D and G). The gene expression changes were generally greater in decedents than survivors at all the time-points when compared to the respective day − 1 paired baseline values (Supp. Figure 4C-H). At D3, we found 892 genes were differentially overexpressed in survivors but at D7 the number of differentially overexpressed genes had reduced to 312 in the survivors. In comparison, in the decedent animals, 958 genes were differentially upregulated at D3, and at D7, we found 710 genes were still upregulated (Supp. Figure 4D, E). These differences were more pronounced for the downregulated genes. While 1513 genes were differentially downregulated in decedents and 1238 genes were differentially downregulated in survivors at D3, at D7, 1452 genes were downregulated in decedents and only 141 genes were differentially downregulated in survivors (Supp. Figure 4G, H). These results suggest that gene expression changes in the surviving animals observed at day 3 normalizes toward D-1 values by D7 but in the decedents, the gene expression changes observed at D3 continue to be observed at D7. This suggests that animals unable to begin resolving the radiation-induced damage by seven days post-exposure did not survive to day 45. Supplementary Tables 3 and 4 list all the differentially regulated genes at all time-points in decedents and survivors, respectively. Supplementary Fig. 5 shows the fifteen most differentially expressed genes exclusively regulated in decedent animals. The microarray expression values of these genes were not statistically significant in the surviving animals across time.

**Table 1:** Stepwise summary of the microarray data.

Filtering criteria	All doses	1.7 Gy	1.9 Gy	2.1 Gy	2.3 Gy	Survivors	Decedents
Repeated measures ANOVA	7485	4673	976	7699	6104	7535	5224
| FC |> 2	2253	2967	452	3437	3595	2373	3021

Data were analyzed along all doses together, and at each dose and survivors and decedents separately. Repeated measure ANOVA and Benjamini Hochberg correction were applied for each variable. Data were further filtered based on ‘Present’ Flags in at least one condition; absolute fold change cut-off was set at 2.

### Ingenuity pathway analysis (IPA) predicted pathways and functions differentially regulated in survivors versus decedents

**Figure 5:**
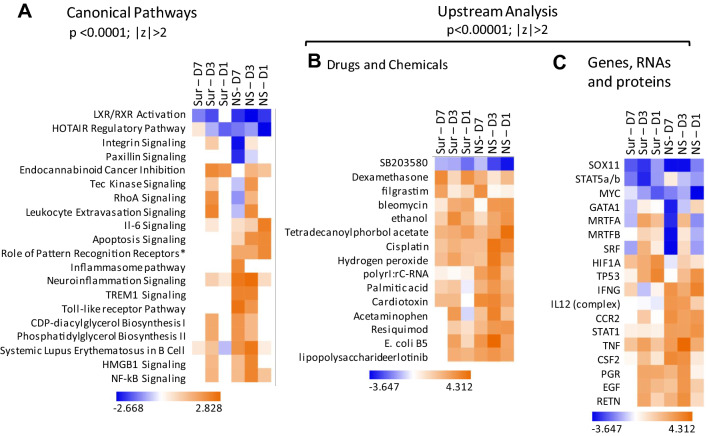
IPA comparison analysis predicted canonical functions and pathways differentially affected in survivors vs. decedents. Heat maps showing (**A**) canonical pathways and (**B**, **C**) Upstream Analysis terms significantly differentially activated (red) or repressed (blue) in any of the conditions. Hierarchical clustering was applied to both pathways and samples. The filtering criteria are shown on the clusters. Sur and NS stand for ‘survivors’ and ‘non-survivors or decedents’ respectively. Sur-D1 stands for genes differentially expressed at day 1 compared to baseline values in survivors; NS-D1 stands for genes differentially expressed at day 1 compared to baseline in decedents; similarly, the remaining labels could be read.

To predict how the observed gene expression changes would translate into altered signaling pathways, we performed a comparison analysis between the survivors and decedents in IPA. The differentially expressed gene-lists meeting the two-fold cut-off at all the time-points for survivors and decedents were uploaded into IPA. The human, mouse and rat gene symbols were employed for analysis since IPA does not support *Sus scrofa* annotations. HOTAIR regulatory pathway, LXR/RXR activation signaling, several immune function-related pathways such as Il-6 signaling, inflammasome pathway, Toll-like receptor pathway and cell cytoskeleton-related pathways such as integrin signaling, paxillin signaling and RhoA signaling were found to be significantly altered in decedents compared to survivors at day 7 (Fig. 5A). HOTAIR regulatory pathway, LXR/RXR activation, integrin signaling and paxillin signaling were predicted to be repressed in the decedents at day 7 while no effect on these pathways was observed at day 7 in survivors. Indeed, several genes belonging to these pathways were differentially expressed (Supp. Figure 6). Inflammasome pathway was activated only at day 7 in the decedents (Fig. 5A). Genes belonging to the inflammasome pathway that were induced in decedents at day 7 and/or day 3 were *IL18*, *TLR4*, *NOD2*, *P2RX7* and *IL1B* (Supp. Figure 6E).

We also performed ‘Upstream Analysis’ in IPA to identify molecules and/or proteins and RNAs which would induce similar gene expression responses. DNA damage-inducing and anti-inflammatory drugs such as cisplatin, hydrogen peroxide, dexamethasone and immune-activating molecules such as poly rI:rC-RNA, E. coli B5 lipopolysaccharide etc. were identified as upstream activators based on the gene expression profiles (Fig. 5B). Gene products such as *TP53*, *IFNG*, *IL12*, *STAT1*, *TNF*, *EGF* etc. were also identified as upstream activators based on the gene expression profiles (Fig. 5C).

### Identification of time- and dose-agnostic gene expression signature for predicting survival

To identify survival dependent gene expression signatures, machine-learning algorithms were employed. Data from all the animals irrespective of dose and time-point was divided into survivor and decedent sets. Baseline expression values from D-1 were not included in the analysis.

Recursive feature elimination (RFE) was used to down select from over 10,000 genes to just 6 genes. At each iteration, a random forest classifier was fit to predict minipig survival status. Up to 15% of the genes were removed based on estimated feature importance in the random forest classifier. A separate RFE procedure was implemented for subject-wise and record-wise cross validation, resulting in two series of gene sets. Radial kernel support vector machines (SVM) were then fit to each gene set in each series, using the appropriate cross validation method for each series respectively.

**Figure 6:**
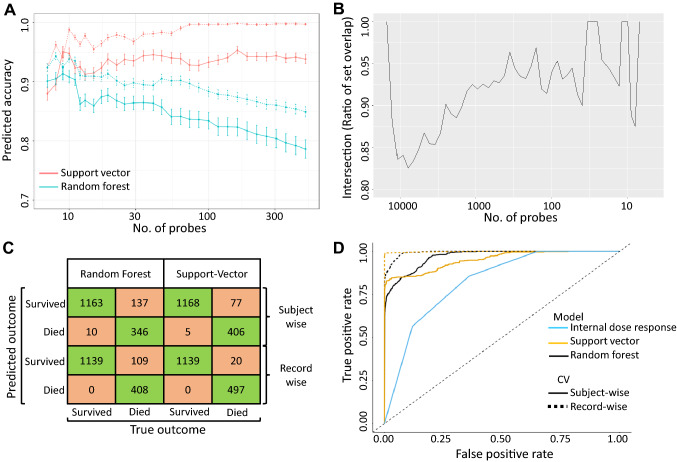
Identification of survival-predictive gene expression signatures using machine learning algorithms. (**A**) Accuracy as a function of the number of genes in the model. Genes were selected by recursive feature elimination (RFE) procedure. Solid and dashed curves are accuracy estimates based on subject-wise and record wise cross-validation procedures, respectively. The blue curves show accuracy of the random forest models that were used in the RFE. The red curves show accuracy estimates for a radial kernel support vector machine using the same genes from the random forest-based RFE. (**B**) Plot showing percent intersection of RFE selected genes between the record-wise and subject-wise cross-validation models across models with varying numbers of genes. (**C**) Confusion matrices based on the most accurate random forest and support vector machine classifier within each cross-validation procedure. Values in the cells show the frequency with which predicted outcomes overlapped with true outcomes. The frequency values were pooled across all 276 individual splits used during the cross-validation procedure; 276 splits each containing 6 observations in the test set result in 1,656 total predictions made. Green colored cells are correct predictions, while orange colored cells are wrongly predicted outcomes (**D**) ROC curves for each model and cross-validation procedure compared to the traditional dose–response. The true positive rate is plotted as a function of the false positive rate, based on a varying threshold for predicting survival status; a positive label corresponds to an outcome/prediction of death. The ROC curves were built using the most accurate model for each classifier and cross-validation procedure. The dose–response was assumed to be a monotonic univariate function of dose, such as a logistic or probit regression model. Only four doses were used in this experiment and, given a threshold probability value, all subjects at that dose would have the same prediction using a dose–response model. Therefore, any dose–response model would have the same ROC curve in this data set. Solid and dashed lines correspond to subject-wise and record-wise cross-validations, respectively. Black and orange lines correspond to Random Forest and Support Vector based classifier, respectively. Blue line represents dose-predictive model and dashed line at 45° represents the random estimate.

Figure 6A shows the accuracy over the series of gene sets obtained from the RFE procedures, split by cross-validation type and model type. The accuracy estimates from record-wise cross-validation procedures were equal to or greater than those obtained from subject-wise cross-validation for both the SVM and the Random forest algorithms across the range of gene sets. The gap between the record-wise and subject-wise cross-validation accuracy is due to the overfitting and underfitting that occur in each procedure, respectively^31^. Therefore, the subject-wise accuracy is a more conservative, downwardly biased accuracy estimate.

Until the number of genes is below 8, the SVM model is more accurate than the random forest, although the random forest model was used for the RFE procedure. Interestingly, for sets with fewer than 100 genes, both models show a peak in subject-wise cross-validation accuracy at 9 genes, with ~ 95% and ~ 91.5% for the SVM and random forest models, respectively. The most accurate model with under 100 genes for each model and cross-validation type was used for subsequent characterization. The most accurate subject-wise cross validation models relied on 9 genes while the record-wise cross validation models relied on 10 genes (Table 2). Although the record-wise and subject-wise cross-validation methods differed in accuracies, a high level of overlap was observed between the two for the most accurate models (nine of the ten genes are common) (Fig. 6B and Table 2). Also, for all the other models, a > 80% intersection was observed between the subject-wise and record-wise cross-validation methods (Fig. 6B).

**Table 2:** The most accurate gene-based classifiers for survival prediction.

Probe name	Gene name	Subject-wise	Record-wise
A_72_P678820	*NEDD9*	1	1
A_72_P599358	*HOPX*	1	1
A_72_P498635	*ECHS1*	1	1
A_72_P400098	*DNAH11*	0	1
A_72_P357313	*BUD13*	1	1
A_72_P284394	*TFIP11*	1	1
A_72_P262634	*TRIM2*	1	1
A_72_P241652	*DPF3*	1	1
A_72_P233607	*PGM1*	1	1
A_72_P015636	*NUAK2*	1	1

Subject-wise most accurate classifier comprised of nine probes and the most accurate record-based classifier comprised of ten probes. As evident, nine of the ten genes were common between the two methods “0” stands for absence and “1” stands for presence in the classifier.

### Model performance characterization

Figure 6C shows the survival/death classification performance for the most accurate model (Table 2) for each cross-validation and model type. The total mortality rate in the experiment was 30% (21/72 data points came from decedents). Because of low sample size, the imbalance in the data propagates into the model predictions creating bias toward predicting a survival outcome. However, the true positive rate (sensitivity; survival is the negative outcome) of the subject-wise random forest and support vector models were 71% and 84%, respectively, which compares well to an expected true positive rate of 30% for a random 70/30 biased guess. Note that the present models consider the outcome of death as the “positive” class, much like in traditional dose–response where probability of mortality or illness is predicted.

The tolerance for false positive and false negatives may depend upon the details of the radiation exposure scenario. Therefore, we constructed receiver operating characteristic (ROC) curves foreach model to explore performance across a range of prediction thresholds. Figure 6D shows that all predictive models perform better than both a random guess (diagonal line y = x) and a dose–response curve (in blue). As stated previously, the record-wise cross-validation results in overfitting and that is clear in the ROC curves. However, the subject-wise cross-validated models which are more conservative estimates of accuracy, also performed reasonably well.

## Discussion

The present study is part of an effort to develop minipigs as improved large animal models for conducting radiation biodosimetry and radiation mitigation studies owing to their physiological response closely resembling that of humans. We investigated the gene expression profiles from blood samples of total-body irradiated minipigs. The doses chosen for the study were previously estimated to correspond to LD_10/45_ (1.7 Gy), LD_25/45_ (1.9 Gy), LD_50/45_ (2.1 Gy) and LD_75/45_ (2.3 Gy). The observed LD values for 1.7 Gy, 1.9 Gy, 2.1 Gy and 2.3 Gy corresponded to LD_0/45_, LD_17/45_, LD_33/45_, and LD_66/45_, respectively, in this study. These differences could be due to the smaller sample size used in the present study, although the observed values do not greatly differ from the estimated literature values.

We observed a time-dependent decrease in the WBC, RBC, platelet and neutrophil counts in both decedents and survivors with a significantly steeper decline observed in the decedents prior to unscheduled euthanasia. However, up to day 7 after irradiation, the observed decline was similar in decedents and survivors. A time-dependent increase in the lymphocyte:neutrophil ratio was observed in decedents till days 14 to 28 but not in the survivors. On the other hand, gene expression profiles of decedents and survivors differed considerably at all the three time-points, day 1, day 3 and day 7. These results indicate that even though the cellular composition of blood is not quantitatively different in the survivors and decedents, gene expression in the whole blood has been differentially altered in decedents compared to survivors.

These gene expression differences also translated into differently regulated pathways in decedents compared to survivors. Notably, lipid metabolic pathways were differentially regulated between survivors and decedents. These differences were most profound at day 7 post-irradiation (Fig. 5A). It was first reported several decades ago that ionizing radiation alters lipid metabolism^32^. We also reported altered lipid metabolism in our recently published findings^29^. Indeed, altered lipid metabolic pathways are a limiting factor in the use of radio and chemotherapy for cancer treatment^33^ and are being investigated as potential therapeutic targets in cancer treatment and cure^34^. Future research encompassing targeting of these pathways through drugs and/or dietary changes would be very informative. Not surprisingly, immune-related pathways were also differentially regulated between survivors and decedents. Inflammation inducing pathways such as IL-6 pathway, Inflammasome pathway, Toll like receptor pathway, leukocyte extravasation signaling, and TREM1 signaling pathway were highly activated in decedents at day 3 and day 7. Few of these pathways were observed to be activated at day 3 in survivors too but not at day 7. These findings suggest a greater unresolved inflammatory response which persisted in decedents even at day 7 and could have possibly contributed to the mortality of these animals. DNA damage-inducing agents and genes and proteins belonging to DNA damage repair pathways were identified to be deregulated in survivors and decedents but to a greater extent in decedents (Fig. 5B,C). These results suggest that both survivors and decedents mount an anti-radiation response, and the response observed in decedents is greater. Indeed, greater number of gene expression changes were observed in decedents compared to survivors at day 3 and day 7 post-radiation (supplementary Fig. 4). All these findings together suggest that decedents sustained higher radiation-induced damage very early on and hence need to orchestrate a more robust response. But even the enhanced response failed to counter the damage radiation inflicted on these decedents. Recognizing that this is a model system, it is reasonable to propose that it may be possible to build survival predictive classifiers in humans as early as one day from irradiation based on gene expression differences for conducting radiation biodosimetry, provided radiation induced changes are observed across species.

Indeed, we show that survival-predictive models could be built from the gene expression profiles using machine-learning algorithms. Highly accurate models comprising nine and ten genes for subject-wise and record-wise cross-validation, respectively, achieved high sensitivity for both random forest and support vector classifiers. Although the models showed a bias towards ‘survival’ prediction because of higher number of survivors compared to decedents, all the models fared better in comparison to a regular dose prediction model achieving high true positive rates (decedent outcomes).

Dose–response curves are the current standard for assessing risk due to radiation exposure. The results presented here clearly demonstrate that a 9/10-gene mRNA biomarker outperforms dose–response in our minipig dataset (Fig. 6D). The predicted performance of a dose–response model would depend on the actual doses received in each scenario and cannot be broadly generalized. Yet, our results show that the identified and refined complex biomarker array discovered in this study (although simple relative to the space of all possible biomarkers) has greater power to assess risk due to radiation exposure compared to a dose–response model.

The ten genes creating the signature are *NEDD9* (Neural precursor cell expressed, developmentally down-regulated 9), *HOPX* (HOP homeobox), *ECHS1* (Enoyl-CoA Hydratase 1), *DNAH11* (Dynein Axonemal Heavy Chain 11), *BUD13* (BUD13 homolog), *TFIP11* (Tuftelin interacting protein 11), *TRIM2* (Tripartite motif containing 2), *DPF3* (Double PHD fingers 3), *PGM1* (Phosphoglucomutase 1) and *NUAK2* (NUAK family kinase 2). NEDD9 also known as HEF1 (human enhancer of filamentation 1) is a scaffold protein and apoptosis mediator (PMID: 10866674). HOPX has been shown to play roles in cardiac development and has tumor suppressive effects in lung cancers (PMID: 15790958, PMID: 17417779, PMID: 12297045). ECHS1 deficiency is associated with mitochondrial fatty acid oxidation disorders (PMID: 29882869). DNAH11 gene variants have been associated with congenital heart disease and are involved in respiratory cilia motility (PMID: 26729821, PMID: 22184204, PMID: 31040315). BUD13 polymorphisms have been associated with elevated serum lipid levels and metabolic syndromes (PMID: 24989072, PMID: 28659142). TFIP11 has been shown to be involved in spliceosome disassembly and is required in enamel development (PMID: 19165350, PMID: 29163197). TRIM2 has tumor-promoting roles in osteosarcoma and colorectal cancer (PMID: 30066883, PMID: 30916596). The histone reader protein DPF3 has been shown to induce proliferation of breast cancer cells (PMID: 31076105). PGM1 is involved in glycogen metabolism (PMID: 30982613). NUAK2 has been shown to be a target of YAP in liver cancer and is actively required for YAP driver growth (PMID: 30446657). Interestingly, of all these genes only NEDD9 has previously been associated with P53 and radiation (PMID: 26011298 and PMID: 19139817). These observations suggest that survival-associated signatures need not bear association with known P53 and radiation responses and it would be imprudent to restrict exploration to known radiation targets. We also observed induction of classical P53 targets such as CDKN1A and MGMT at day 1 post-radiation (Fig. 4). The other differentially expressed genes were VPREB3, MS4A1, CD72, CD79A, A2M and SFXN3. Genes differentially expressed exclusively in the decedents included FN1, MELK, RSC1A1, ANG, ELL3 and FAM212B (Supplementary Fig. 5).

The technology used in the study could not be employed in a mass causality scenario. It requires sophisticated instrumentation and expert teams for data generation and analysis. The output signals differentiating survivors and non-survivors could however be transformed and adapted to a field deployable point-of-care device. Further studies investigating the usability of the signatures identified here on a different format are warranted. It might be possible that in the near future hand-held sequencing devices, which could perform all the steps starting from RNA isolation to data analysis, will become common and affordable^35,36^ and could be easily adapted for radiation biodosimetry triage.

It is necessary to investigate if differences observed in Gottingen minipigs are present in other higher mammals such as non-human primates. A cross-species presence would suggest humans would have similar alterations in response to radiation; hence these differences could be used as biomarkers and/or therapeutic targets. Few studies have indeed succeeded in translating gene expression signatures from animal models into humans although expression values needed to be adjusted to human applicable scales^7,37,38^. Immune related pathway changes identified in minipigs in our report were also observed after whole thorax irradiation in non-human primates by Gandhi et al^39^. This is an encouraging similarity. The limitations of minipig models are that genome is not well annotated and much still needs to be learned. As we keep learning more about them, it should be possible to effectively translate these signatures into humans.

The implications of this study include a potential paradigm shift such that triage and medical decision making following total-body or extensive partial-body radiation exposure can be done based on a predicted outcome. Currently, there is the potential for confusion in that the unit of biodosimetry is the Gray, which is the same unit for physical dosimetry. Coleman and Koerner sought to distinguish them^1^ by using the term “biodose” wherein “*Biodose* is a measurement obtained by exposing living systems, albeit cells, tissues or animals, to a known amount of radiation (dose) and examining a biological change, which might be a chromosomal, molecular, proteomic or other physiological response”. At the time of exposure, the biological change is measured and related back to a dose (Gy). While “*Dose* is a physical measure of radiation absorbed, the Unit is Gray which is the absorption of one joule of energy per kilogram of matter.” When a biodose is provided to a decision-maker it will be an estimate of the physical dose which relates to the probability of lethality, usual over a 30-day period for the ARS. For example, an LD_50_ is a dose at which half the victims would die. Thus, it assigns a “risk group” for survival without treatment that dictates where a person is sent after assessment which might be home, directly to the hospital, or to a location for additional surveillance. An early assay that can predict the likelihood of a consequence would be much better in allocating resources. The medical intervention would be using that information to help the physician determine a course of treatment. Notably, the biodose is a measure that is generally used by the clinician as part of decision-making although a physical dose may also be available from a dosimeter, also in Gy.

In scenarios where treatment resources are not constrained, a higher true positive rate maximizes lives saved by maximizing treatment coverage. However, if resources are constrained then a comparatively lower false positive rate minimizes wasted resources, thereby maximizing the total number of lives saved. Interestingly, the random forest model out-performs the SVM in the high true positive rate regime, but the SVM is superior in the low false positive regime. Therefore, the random forest model would be best for low-casualty scenarios whereas the SVM model would be best for triage scenarios. In the future, it may prove useful to incorporate additional information about biomarkers of treatment efficacy as a function of dose-identification of individuals likely to respond to treatment could improve model performance.

It is possible that not only might a prediction of survival be possible but a recent report from our lab has demonstrated that organ specific biomarkers from the heart, liver and lung tissues can predict survival^29^. The physiological and functional dysfunction from these organs may occur in months (called Delayed Effects of Acute Radiation Exposure (DEARE)) or even many years later. The follow-up period of 45 days for this study and the previous report^29^ was a limitation for studying DEARE. We observed organ-injury in the form of edema and hemorrhage in lungs, heart, GI, kidney, liver and spleen of many animals irrespective of whether they survived or not but DEARE related symptoms such as fibrosis were not found in any of the animals^29^. Possibly, if these animals were followed up for longer period, we could have observed DEARE in survivors. Indeed, in a recent report Hritzo et al., have shown that Gottingen minipigs followed up for 120 days after partial body irradiation did present late health effects such as collagen deposition and fibrosis of the heart and kidney and dysregulated IGF-1 signaling pathway^40^. Besides, liver and kidney marker profiles were also different^40^.

The progression toward improved functional biomarkers requires several steps including further evaluation of the use of serum/plasma markers that may contain information that might not be seen in whole blood. That would then be a secondary analysis for those who are deemed to need treatment. Should it be possible to predict organ specific injury, clinical mitigation could address both ARS and DEARE. The specifics of the clinical assay development would depend on the specific platform, absolute level of the biomarker for detectability and within-assay validation and possibly a decision tree analysis in that the time after the incident will be known such that the utility of a marker or set of markers may vary over time after the incident^20^.

The present study is part of the evolution of radiation biomarkers to assess radiation injury, guide triage and medical management, enable better use of resources during a scarce resource setting and improve the efficiency and effectiveness of a mass casualty response. The use of the organ specific biomarkers might have clinical utility for organ tissue dose and tolerance. This study builds on the investment in capabilities for disaster response from NCI, NIAID, BARDA and DoD in the US to enhance disaster preparedness for mass-casualty incidents.

## Materials and methods

### Animal husbandry and treatment

Five to six-month-old male Gottingen minipigs (*Sus scrofa*) were used in this study. These animals were maintained at 10% genetic variation. All experiments were conducted following relevant guidelines and regulations. The work presented follows ARRIVE guidelines^41^. Animals were housed in humane conditions in an Association for Assessment and Accreditation of Laboratory Animal Care (AAALAC) accredited lab at the Armed Forces Radiobiology Research Institute (AFRRI, Bethesda MD)). The AFRRI Institutional Animal Care and Use Committee (IACUC) approved all animal experiments and all efforts were made to minimize animal suffering. This study was one of several to use specimens from these animals to minimize the number of animals used and maximize the information obtained^29^. Animals were exposed to total-body irradiation using a Cobalt-60 source in the AFFRI Cobalt facility, to a total dose of 1.7, 1.9, 2.1 or 2.3 Gy (n = 6/dose) selected to bracket the anticipated LD_50/45_ (Lethal dose for 50% of the animals). Details of dosimetry have previously been published^29,42^. Briefly, animals were unilaterally sequentially exposed to total midline body doses at a dose rate of 0.5–0.6 Gy/minute. Animals were anesthetized with an IM injection of TelazolVR (100 mg/mL, 2 mg/kg) and Xylazine (50 mg/mL, 1 mg/kg) before placing in a sling for the duration of irradiation. The two sources were raised sequentially with a lateral geometry of exposure. The unilateral sequential exposure is derived from the set-up of the AFRRI Cobalt-60 Radiation Facility which contains two sets of Cobalt-60 rods that are lifted sequentially to generate a field. Dose rates were determined using an alanine/ESR (electron spin resonance) dosimetry system (American Society for Testing and Material Standard E 1607) contained in water-filled cylindrical pig phantoms. Dose rates in phantoms were converted to the dose rate for the animals by accounting for the decay of the ^60^Co source, the small difference in mass energy-absorption coefficients for water and soft tissue, and size of the animal. The radiation field was uniform within ± 2%. Additionally, real time dosimetry of the output dose was measured using an ion chamber system. Day of exposure was considered as day 0. Blood was collected one day prior to exposure (day − 1) and on days 1, 3 and 7 post-exposure directly into RNAeasy Protect Animal Blood Tubes (Cat #76544, Qiagen). These animals were followed up for 45 days. During the 45-day study period, pigs were assessed at least twice daily for signs of pre-established criteria that necessitated unscheduled euthanasia. Prior to euthanasia, anesthesia was induced with 5–2% isoflurane and animals received an IM injection of Xylazine and Telazol (as described above). Euthasol (sodium pentobarbital) was injected IV for euthanasia. Unscheduled euthanasia was necessitated if one absolute criteria (non-responsiveness, dyspnea, loss of ≥ 20% body weight, hypothermia) or four non-absolute criteria (hyperthermia, anorexia, anemia, vomiting/diarrhea, lethargy, seizures/vestibular signs, prolonged hemorrhage) were observed.

### Survival analysis

Survival analysis was performed using Kaplan–Meier method in GraphPad Prism.

### RNA isolation

Total RNA was isolated from the whole blood samples using the RNAeasy Protect Animal Blood Kit (Cat # 73224, Qiagen) according to the manufacturer’s protocol. Quality and quantity of the RNA samples were assessed using a Denovix DS-11 nanodrop spectrophotometer (Denovix, DE, US) and Agilent Bioanalyzer with the RNA6000 Nano Lab Chip (Agilent Technologies, Santa Clara, CA).

### Microarray experiment and analysis

Total RNA was reverse transcribed after priming with a DNA oligonucleotide containing the T7 RNA polymerase promoter 5’ to a d(T)24 sequence. After second-strand cDNA synthesis and purification of double-stranded cDNA, in vitro transcription was performed using T7 RNA polymerase. The quantity and quality of the cRNA was assayed by spectrophotometry on the Agilent Bioanalyzer as indicated for Total RNA analysis. cRNA was fragmented to uniform size and hybridized to Porcine (V2) gene expression microarrays (Design ID-026440, Agilent Technologies). Slides were washed and scanned on an Agilent SureScan Microarray Scanner. Expression values were extracted using Agilent Feature Extraction software and data were analyzed with GeneSpring GX software (Agilent Technologies). Array normalization and batch correction were performed using COMBAT-Quantile R-script in GeneSpring. Data analysis was also performed in GeneSpring. Time-point data from the same animals were analyzed using repeated measures ANOVA. Multiple testing correction was performed using Benjamini Hochberg False Discovery Rate (FDR). Principal component analysis (PCA) plots were generated reducing the data to three principal components.

### Ingenuity pathway analysis (IPA)

Both core and comparison analyses were performed in IPA (Qiagen, US). All the pathways and function terms that satisfied the absolute z-score of more than 2 and p-value of less than 0.00001 were predicted to be altered based on the gene expression data. Since IPA does not support *Sus scrofa* gene annotations, corresponding human, rat and mouse gene symbols were used to predict regulated pathways.

### Signature prediction

Survival dependent signatures were predicted from the gene expression data as follows: Raw data were pre-processed using *limma* package in R^43^. Raw expression values were background corrected with normal-exponential correction. Genes below background in all samples within at least one treatment level were removed. The expression values for the filtered probe set were quantile normalized between sample arrays. Transcripts with multiple genes were averaged such that the final set reflected best estimates of transcript level expression and control genes were removed.

### Cross-validation schemes

Cross-validation is a procedure for obtaining accurate estimates of the performance of a predictive model by iteratively splitting the individual records (i.e. record-wise) into sets for training and testing the model. However, the cross-validation procedure implicitly assumes that the data in the training and testing sets are independent and identically distributed (iid) random samples. The iid assumption is clearly violated in datasets with repeated measures from the same subject, such as the minipig survival dataset in the present study. Creating independence between the testing and training sets is achieved by splitting the data subject-wise^44^, i.e. not allowing data from any subject to be spread across both test and training sets. However, the subject-wise split does not ensure that the testing and training sets are identically distributed, especially when the number of subjects is small, due to potential differences between the subjects^45^. The current best practice is to perform both record-wise and subject-wise cross-validation with the understanding that the record-wise procedure overestimates performance while the subject-wise procedure underestimates performance^31^.

The dataset was composed of 24 subjects with 3 time-point samples from each subject. For subject-wise cross-validation the models were trained on data from 22 subjects and tested on data from the remaining 2 subjects. Only 276 ways exist to choose 2 subjects from 24, making it feasible to test every split. For consistency, a random sample of 276 record-wise splits was performed where 6 individual records were held out for testing.

### Recursive feature elimination (RFE)

Building a predictive model with a data set where the number of samples (72) is much smaller than the number of genes (> 10,000) is problematic. Therefore, some method of regularization was needed to prevent overfitting. Additionally, the number of genes that could be used in a field-deployed radiation biodosimeter is small. Therefore, recursive feature elimination (RFE) was used to identify a small subset of genes that could be used to build an effective predictive model.

RFE is a process to iteratively remove features (genes) that do not contribute substantially to predictive performance. Custom scripts were developed to implement RFE with subject-wise and record-wise cross-validation, because no R package exists for that purpose. At each RFE iteration a random-forest model was fit across a hyperparameter grid and at least 15% of the genes were removed based on low feature importance values in the best fit model. The mean and variance of the accuracy estimate across cross-validation splits was stored for each iteration. The RFE procedure resulted in a sequence of probe sets of decreasing size and associated accuracy metrics. To test the generalizability of the genes, a radial support vector machine (SVM, a machine learning algorithm for data classification) was fit to each probe set. The parameters of the SVM were similarly obtained by tuning across a hyperparameter grid.

### ROC curve analysis

Receiver operating characteristic curves were obtained to characterize the performance of our models. A ROC curve was obtained for each model and cross-validation method. A reference ROC curve was also constructed for a lognormal generalized linear model- (probit) based dose–response curve. No dose–response data was needed to build the dose–response ROC curve because any univariate monotonic function of dose alone would have the same ROC curve given the survival study data set.

## Supplementary Information

Supplementary Information 1.

Supplementary Information 2.

Supplementary Information 3.

Supplementary Information 4.

Supplementary Information 5.

Supplementary Information 6.

Supplementary Information 7.

Supplementary Information 8.

Supplementary Information 9.

Supplementary Information 10.

Supplementary Information 11.
