# Supplementary material for: Whole blood gene expression within days after total-body irradiation predicts long term survival in Gottingen minipigs
Source: Sci Rep. 2021 Aug 5;11:15873. doi: 10.1038/s41598-021-95120-5 (PMC8342483; doi:10.1038/s41598-021-95120-5)
Supplement: Supplementary file 1 — Supplementary Information 1. [file 41598_2021_95120_MOESM1_ESM.docx]

**Supplementary Figure 1: Neutrophil counts as a function of time in decedents and survivors.** A) Neutrophils counts over the 45-day monitoring period, B) neutrophil counts over 7-days immediately following irradiation.

**Supplementary Figure 2: Linear regression analysis showing lymphocyte:neutrophil (L:N) ratio as a function of time in decedents and survivors.** A) L:N ratio over the 45-day monitoring period, B) L:N ratio over 7-days immediately following radiation.

**Supplementary Figure 3: Visualization of the differences in the gene expression profiles at different time points when each dose group was analyzed individually.** PCA plots were generated in GeneSpring using the differentially regulated probe-list passing the filtering criteria (p <0.05; | FC| >2).

**Supplementary Figure 4: Decedent animals display more pronounced gene expression changes compared to the survivors.** A) PCA plot was generated in GeneSpring using the differentially regulated probe-list passing the filtering criteria (p <0.01; | FC| >2) in all the decedents. B) PCA plot was generated in GeneSpring using the differentially regulated probe-list passing the filtering criteria (p <0.01; | FC| >2) in all the survivors. C- H) Venn diagrams depicting overlap between the differentially regulated genes in decedents and survivors at each time point analyzed. C, D, E show overlap in the upregulated genes (red) while F, G, H show overlap in the downregulated genes (blue).

**Supplementary Figure 5: Box and whisker plots of the fifteen most highly significantly regulated genes across animals**. The normalized intensity values for each animal are plotted on y-axis. Data values are represented in a box plot with mean and error bars denote standard deviations. Decedent animals are represented in red and survivors are represented in grey. The microarray expression values of these genes were not statistically significant in the surviving animals across time. Different time-points are shown with increasing intensities of color (lighter- day -1; light- day 1; dark- day 3; darkest- day 7).

**Supplementary Figure 6: Heat maps of genes belonging to A) HOTAIR signaling, B) LXR/RXR Activation, C) Integrin Signaling, D) Paxillin Signaling and E) Inflammasome Pathway predicted repressed in decedents.** Hierarchical clustering was applied to both pathways and samples. Absolute z-score cut-off was 2 and p-value cut-off was 0.00001. Red and green colors signify induced or repressed expression in comparison to baseline values. Blue and orange bars at the top of the clusters signify cumulative effect on the pathway in the specific condition based on the underlying gene expression changes. Sur and NS stand for ‘survivors’ and ‘non-survivors or decedents’ respectively. Sur-D1 stands for genes differentially expressed at day 1 compared to baseline values in survivors; NS-D1 stands for genes differentially expressed at day 1 compared to baseline in decedents; similarly, the remaining labels could be read.

**Supplementary Table 1: Blood pathology parameters recorded at different days in all experimental animals**

**Supplementary Table 2: List of differentially expressed genes at any time-point across all animals irrespective of dose and survival.** Differentially expressed genes were identified by repeated measure ANOVA and Benjamini Hochberg (FDR) correction (p<0.001) with a 2-fold cut-off in comparison to baseline values at day -1. The columns A through H correspond to probe name, corrected p-value, D1 fold change, D3 fold change, D7 fold change, Gene Symbol, Description and sequence spotted on the array for the probe, respectively.

**Supplementary Table 3:** **List of differentially expressed genes at any time-point across all decedents.** Differentially expressed genes were identified by repeated measure ANOVA and Benjamini Hochberg correction (p<0.001) with a 2-fold cut-off in comparison to baseline values at day -1. The columns A through H correspond to probe name, Gene Symbol, Description, sequence spotted on the array for the probe, corrected p-value, p-value, D1 fold change, D3 fold change, D7 fold change, and status in survivors, respectively. P stands for present and A for absent (i.e. whether the gene was also differentially expressed in survivors)’. Fold change > 2 is highlighted in red and < 2 is highlighted in blue.

**Supplementary Table 4:** **List of differentially expressed genes at any time-point across all survivors.** Differentially expressed genes were identified by repeated measure ANOVA and Benjamini Hochberg (FDR) correction (p<0.001) with a 2-fold cut-off in comparison to baseline values at day -1. The columns A through H correspond to probe name, Gene Symbol, Description, sequence spotted on the array for the probe, corrected p-value, p-value, D1 fold change, D3 fold change, D7 fold change, and status in decedents, respectively. P stands for present and A for absent (i.e. whether the gene was also differentially expressed in decedents). Fold change > 2 is highlighted in red and < 2 is highlighted in blue.
