# Supplementary figures and images for: Whole blood gene expression within days after total-body irradiation predicts long term survival in Gottingen minipigs
Source: Sci Rep. 2021 Aug 5;11:15873. doi: 10.1038/s41598-021-95120-5 (PMC8342483; doi:10.1038/s41598-021-95120-5)

A

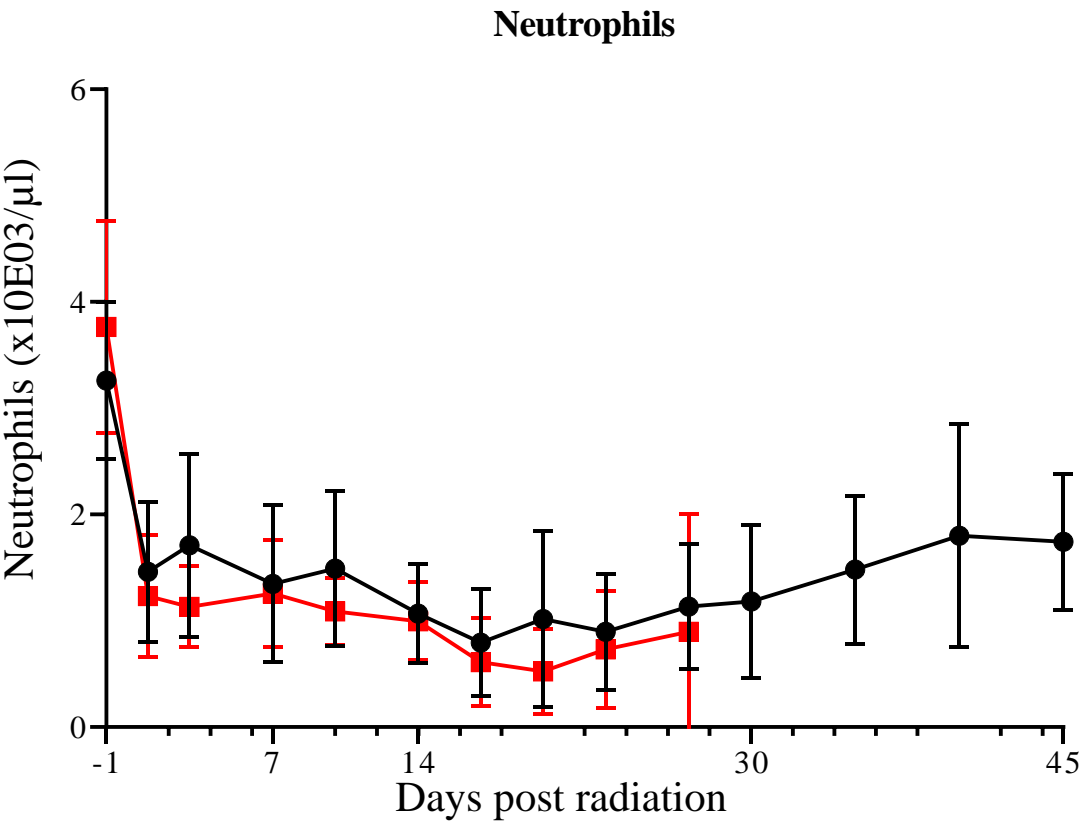

B

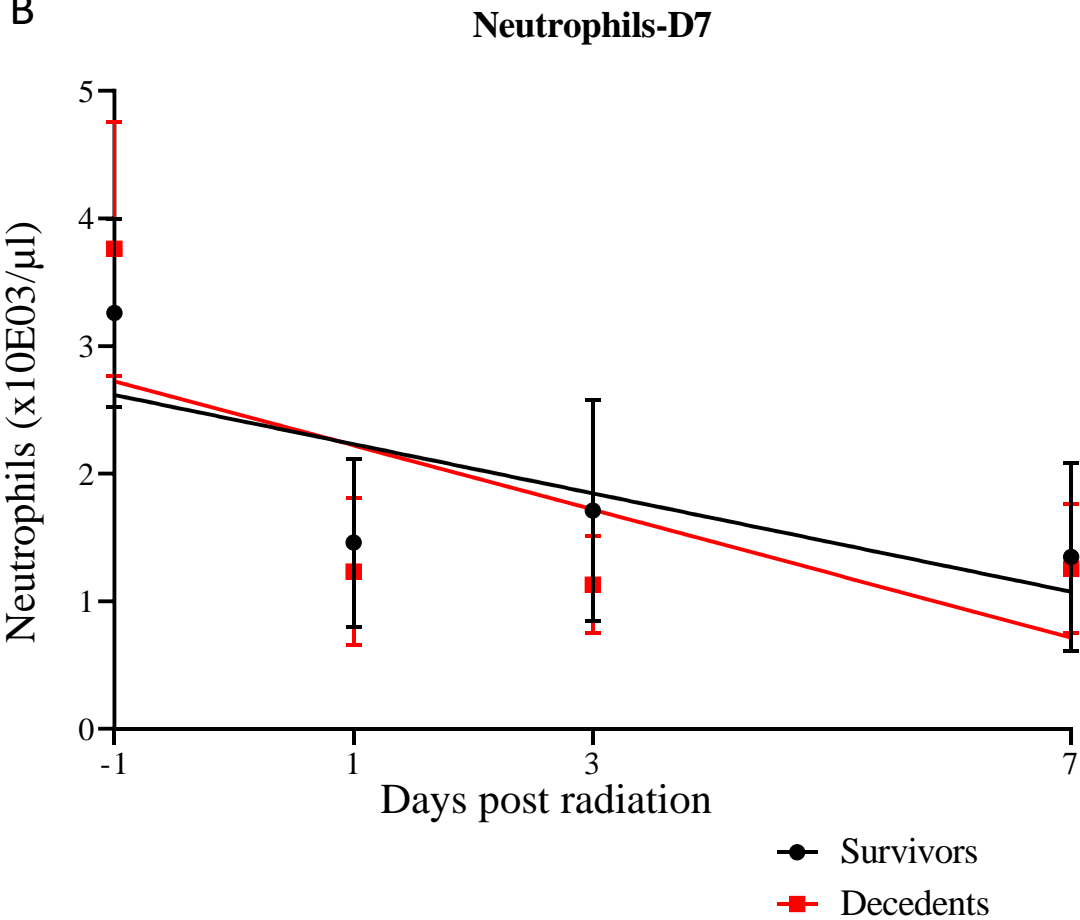

Supplement: Supplementary file 2 — Supplementary Information 2. [file 41598_2021_95120_MOESM2_ESM.pdf]

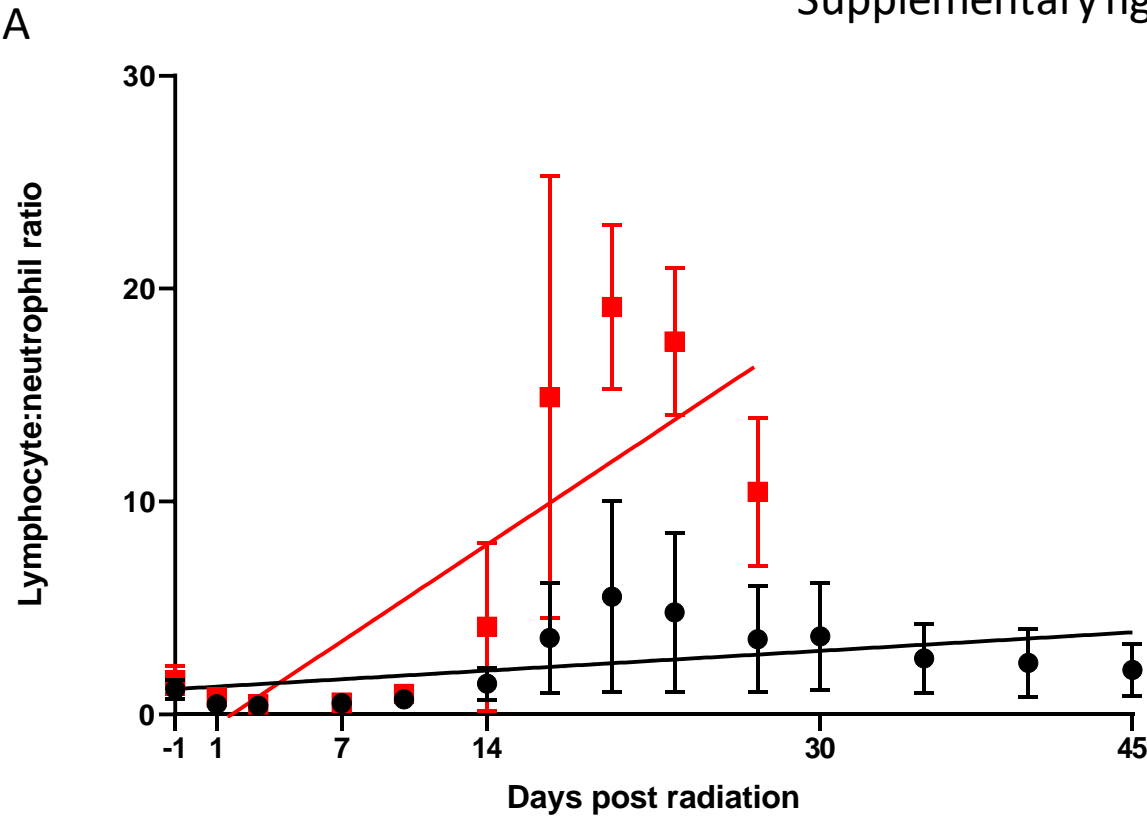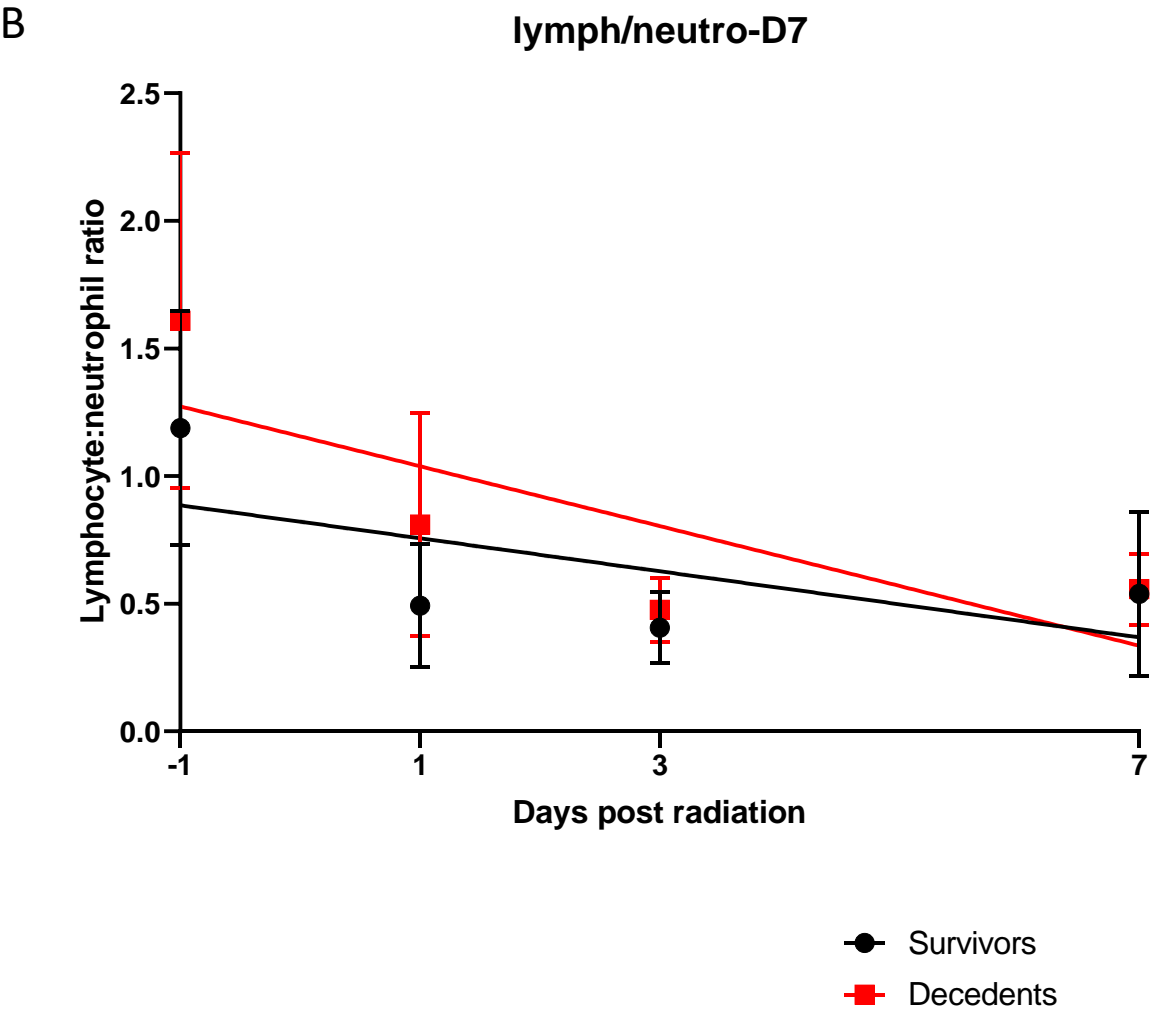

Supplement: Supplementary file 3 — Supplementary Information 3. [file 41598_2021_95120_MOESM3_ESM.pdf]

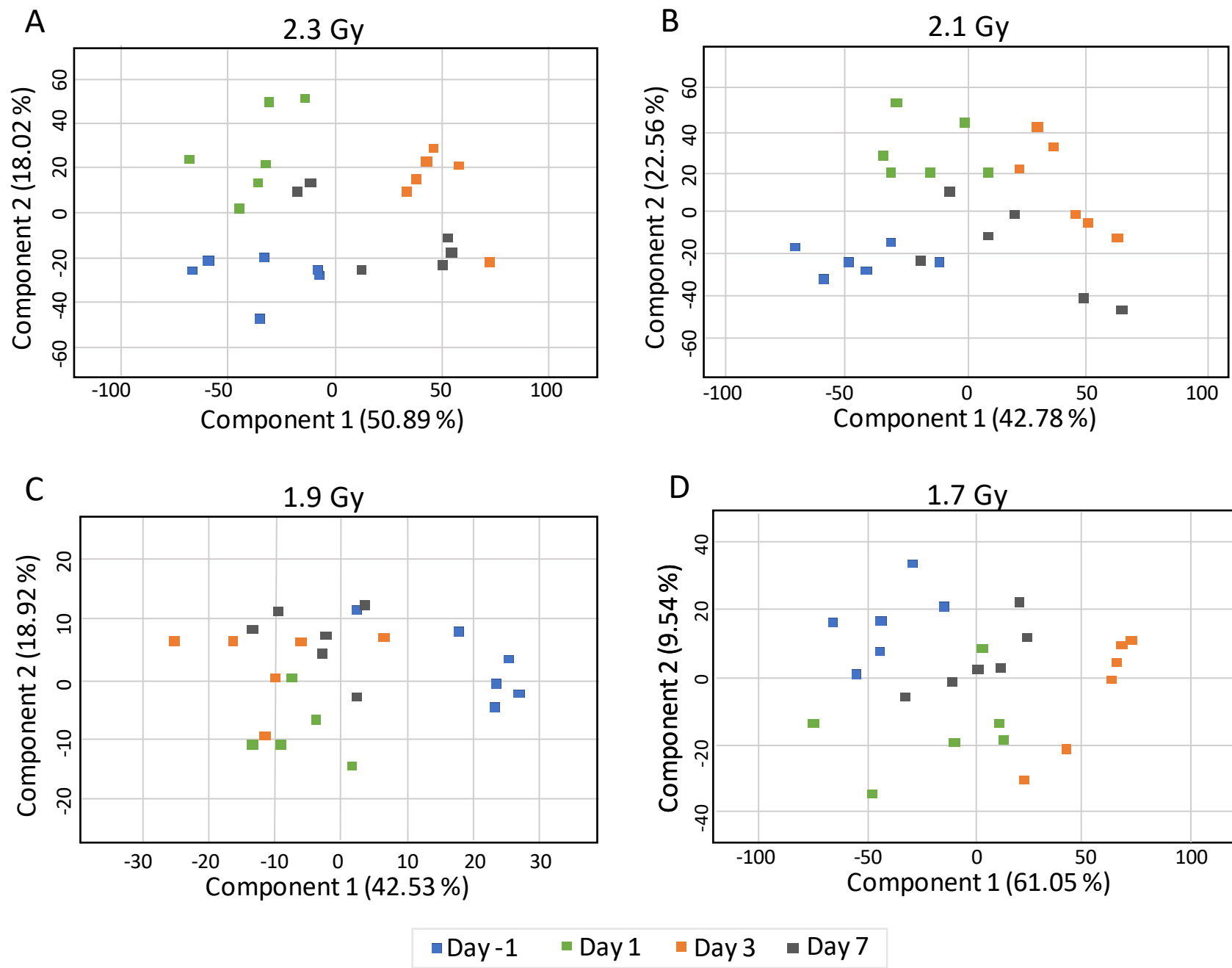

Supplement: Supplementary file 4 — Supplementary Information 4. [file 41598_2021_95120_MOESM4_ESM.pdf]

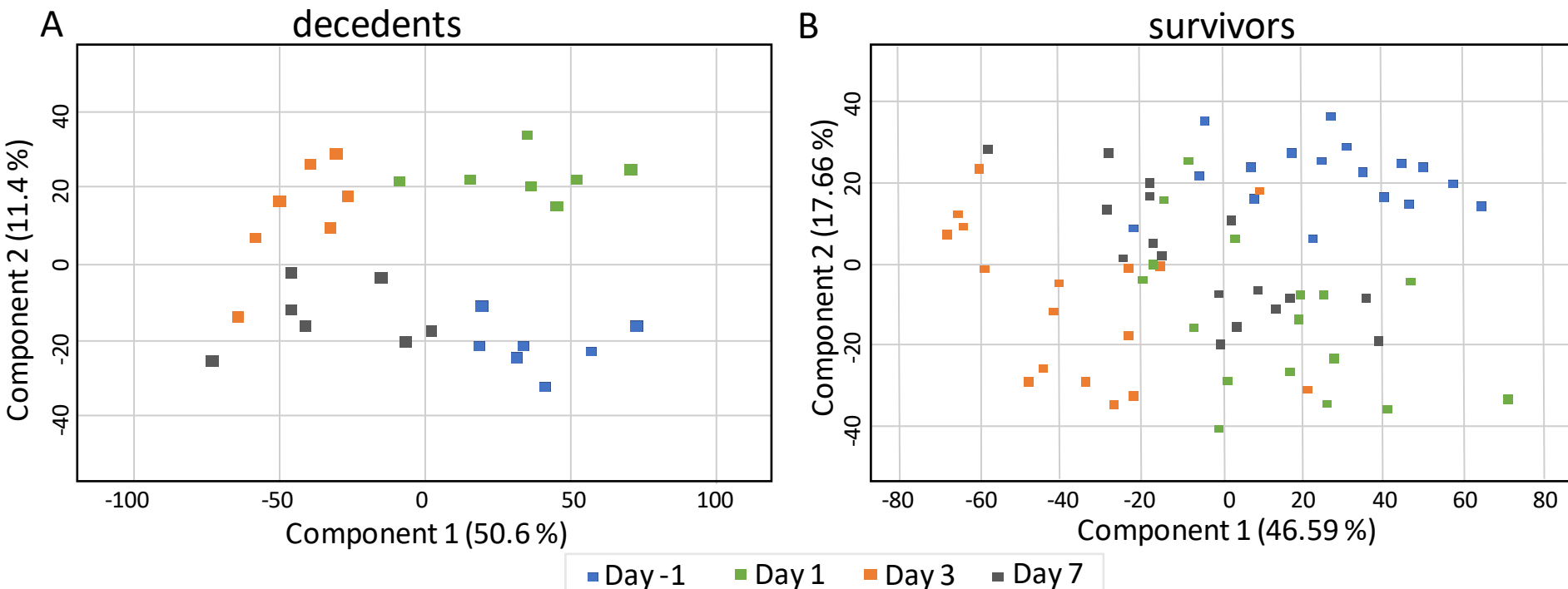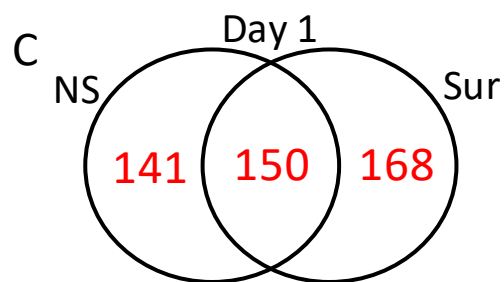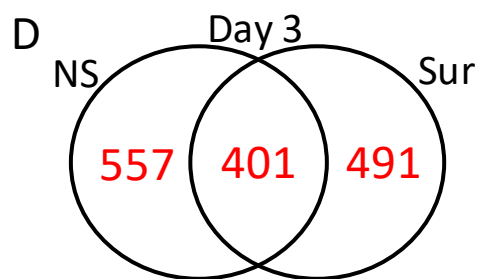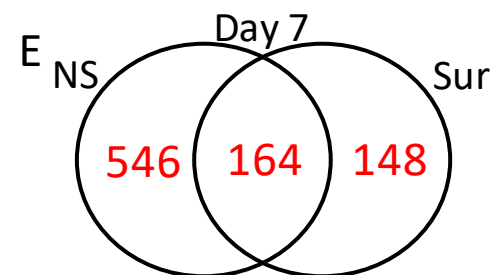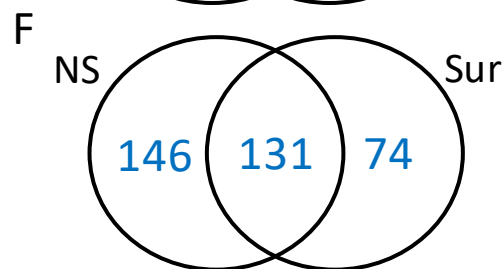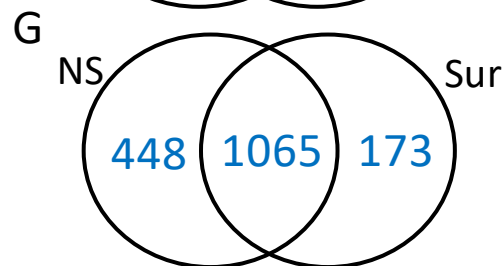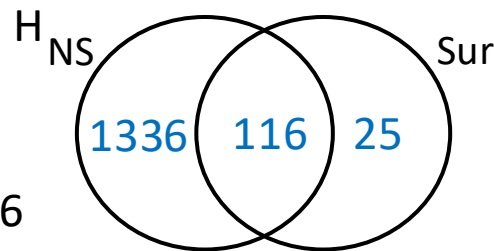

986

Supplement: Supplementary file 5 — Supplementary Information 5. [file 41598_2021_95120_MOESM5_ESM.pdf]

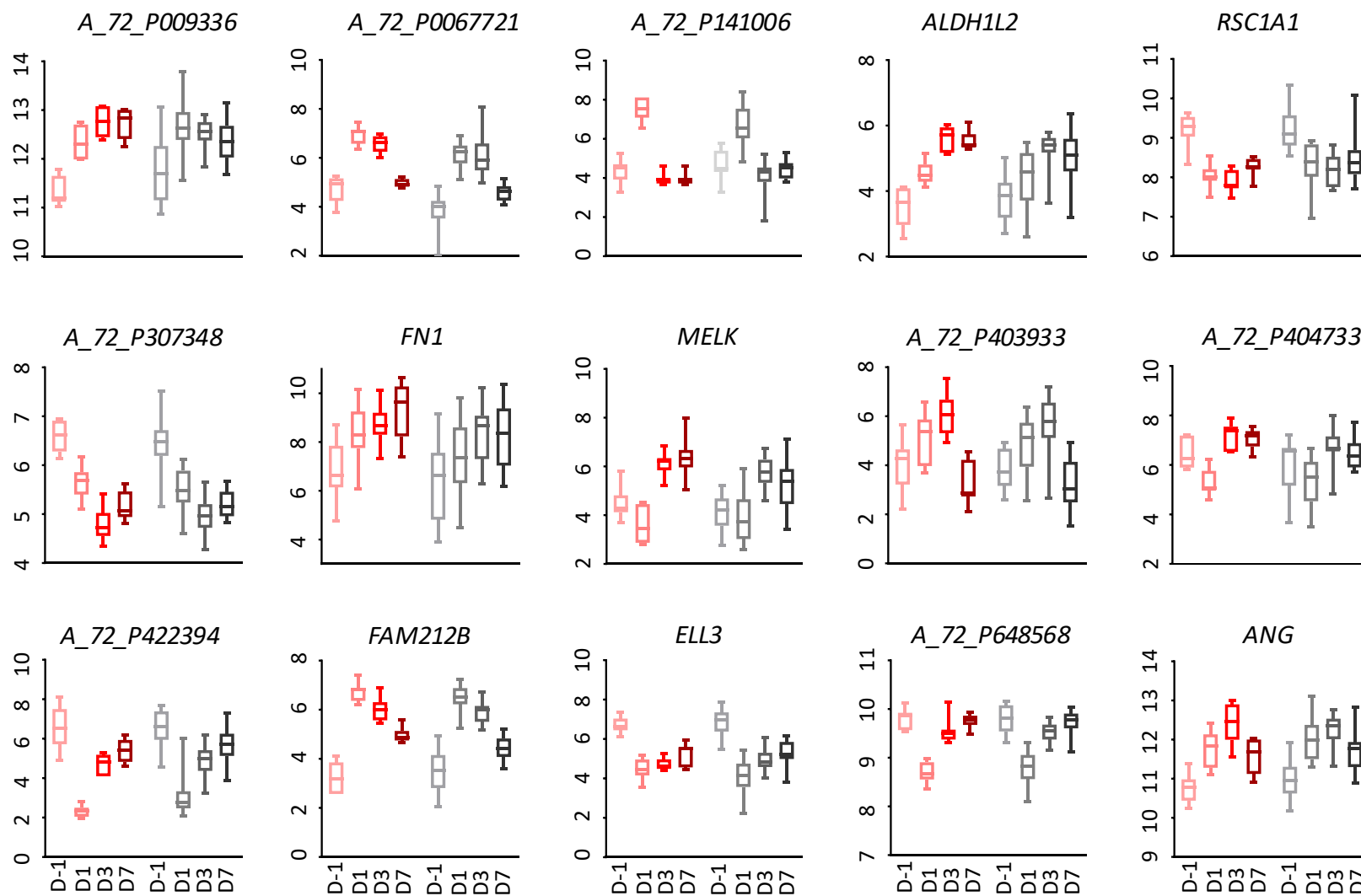

Supplement: Supplementary file 6 — Supplementary Information 6. [file 41598_2021_95120_MOESM6_ESM.pdf]

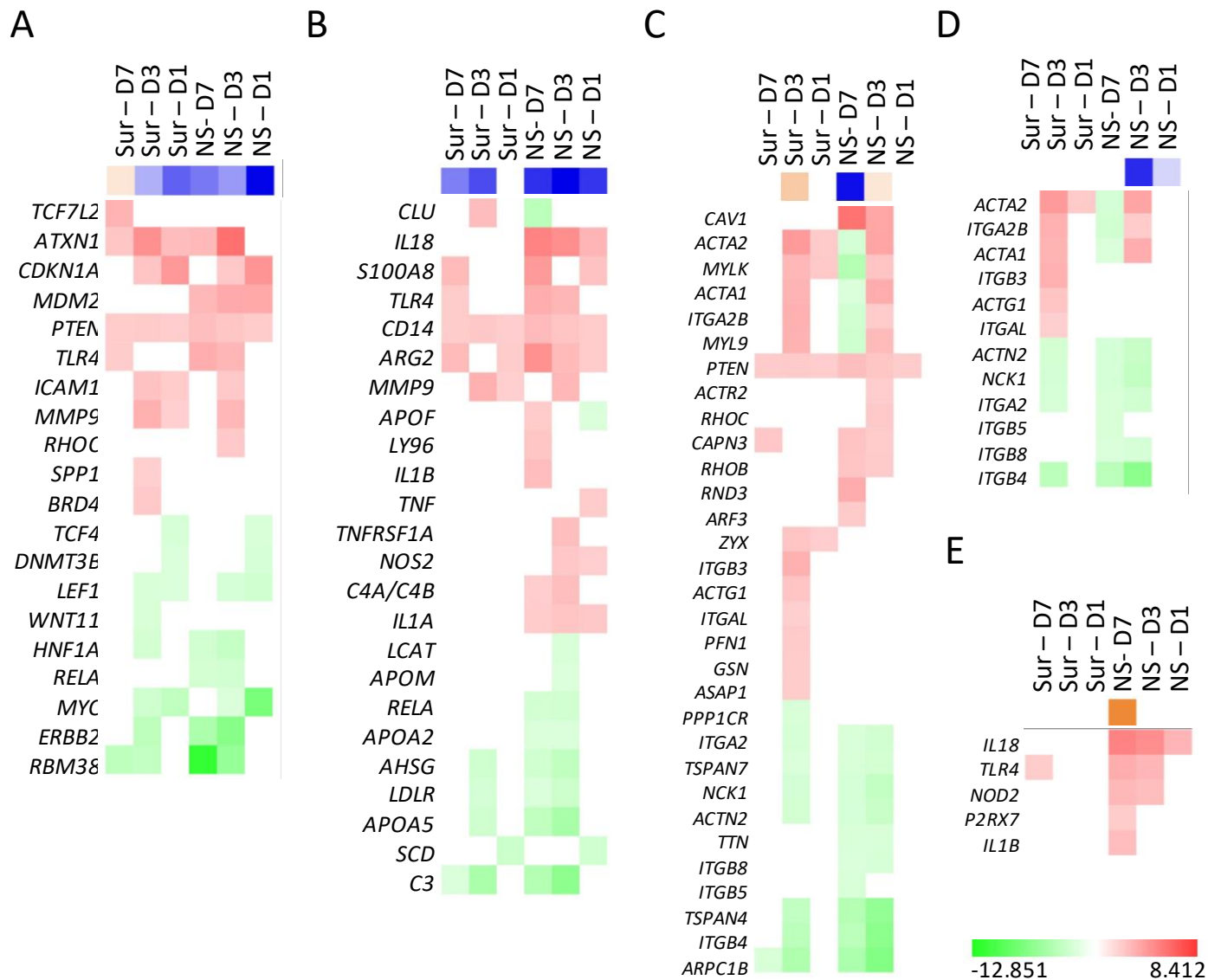

Supplement: Supplementary file 7 — Supplementary Information 7. [file 41598_2021_95120_MOESM7_ESM.pdf]
